# Diagnostic accuracy of fetal cardiac magnetic resonance imaging in cardiac and thoracic vascular anomalies: a meta-analysis

**DOI:** 10.1186/s12880-026-02521-x

**Published:** 2026-06-23

**Authors:** Chandana Bhargavi, Rathika Damodara Shenoy, Adrian Rassaf, Daiki Jonouchi, Elvis Anunwa, Suchetha S. Rao, Nutan Kamath, Juan M. Acuna

**Affiliations:** 1 Department of Anatomy and Medical Imaging, American University of Antigua College of Medicine, Osbourn, Antigua and Barbuda; 2 Department of Pediatrics, Kasturba Medical College Mangalore, Manipal Academy of Higher Education, Manipal, India; 3 Department of Clinical Medicine, American University of Antigua College of Medicine, Osbourn, Antigua and Barbuda

**Keywords:** Aorta, Cardiac anomalies, Complex congenital heart disease, Hierarchical models, Prenatal, Random effects model

## Abstract

**Background:**

Fetal echocardiography (FE) is the primary modality for screening cardiac anomalies. Diagnostic limitations persist in complex lesions, thoracic vascular anomalies, late gestation, and cases with poor acoustic windows. Fetal cardiac magnetic resonance imaging (FCMRI) complements FE by providing additional anatomical details of cardiac and thoracic vascular anomalies, although its diagnostic test accuracy has not been systematically evaluated. This meta-analysis aimed to determine the diagnostic accuracy of FCMRI in detecting cardiac and thoracic vascular anomalies and to evaluate its potential role in prenatal decision-making.

**Methods:**

Observational studies evaluating FCMRI for cardiac and thoracic vascular anomalies were analyzed at the lesion-description level. Summary statistics were estimated using a bivariate random-effects and hierarchical summary receiver operating characteristic models. Subgroup analyses were performed across anatomical lesion categories, image acquisition methods, and acquisition eras.

**Results:**

Seven studies involving 449 cardiac and thoracic vascular anomalies were analyzed. FCMRI demonstrated a sensitivity of 0.80 (95% CI: 0.66–0.89) and specificity of 0.98 (95% CI: 0.92–0.99), indicating acceptable accuracy, with a 95% prediction ellipse area of 0.29, suggesting moderate between-study heterogeneity. Pooled sensitivity [0.88 (95%CI: 0.79–0.93)] was highest for complex heart defects, with a tightly clustered prediction ellipse (0.04). Thoracic vascular anomalies showed lower sensitivity [0.75 (95%CI: 0.56–0.88)] and greater heterogeneity (0.39), reflecting diagnostic challenges. Only two studies showed incremental clinical utility of FCMRI in decision-making that varied by specific cardiac and thoracic vascular anomaly. The analysis was limited by the small number of studies, methodological heterogeneity, and the underrepresentation of true-negative cases.

**Conclusions:**

Fetal CMRI can provide clinically useful anatomical information complementary to FE, particularly in late gestation and selected high-risk pregnancies. Although it demonstrated high specificity and moderate sensitivity overall, diagnostic performance varied across lesion subtypes, with aortic and aberrant vascular anomalies remaining the most challenging to diagnose prenatally.

**Meta analysis registration:**

PROSPERO: CRD420251133115.

**Supplementary Information:**

The online version contains supplementary material available at https://doi.org/10.1186/s12880-026-02521-x.

## Introduction

Screening for isolated congenital heart disease (CHD) during pregnancy is the standard of care for all women. CHDs are the most common birth defects; the prevalence at birth is approximately nine per 1,000 live births, higher among fetuses, as pregnancy losses occur in one-third [1, 2]. Despite progress, the diagnosis of cardiac and thoracic vascular anomalies (CTVAs) remains imperfect, with only about 80% of affected fetuses detected. Prenatal early detection enables syndromic diagnosis, risk stratification, improved counseling, and allows for optimal perinatal management [3]. Universal obstetric ultrasound between 18 and 22 weeks of gestation is the standard of care for fetal malformation screening and diagnosis. High-risk pregnancies, such as those with a family history of CHD, maternal diabetes, or abnormal findings on ultrasound, are referred for detailed fetal ultrasound, including fetal echocardiography (FE), the primary modality for prenatal cardiac assessment [3–5]. 

A meta-analysis showed high diagnostic test accuracy (DTA) of FE for CHD detection using the first-trimester four-chamber, outflow tracts, and three-vessel trachea views (4CV, OTVs, and 3VT) protocol. The pooled sensitivity and specificity were 0.84 and 1.00 respectively [6]. However, malformations requiring progressive, continuous measurement assessment, such as aortic diameter in coarctation of the aorta (CoA), or for smaller, harder-to-assess defects, such as an anomalous pulmonary venous connection, are often missed [7, 8]. These intrinsic, defect-specific diagnostic limitations become more challenging when suboptimal acoustic windows are present, such as in late gestation, maternal obesity, oligohydramnios, adverse fetal lie, or bony shadowing.

FCMRI is indicated as a complementary, second-line diagnostic modality to enhance inconclusive FE diagnoses or when improved anatomic delineation is required [3]. Initial attempts at FCMRI began in 1984 using 0.15–0.35 Tesla (T) systems; however, image resolution was limited by fetal motion and small cardiac size [9]. By 2005, techniques such as T2-weighted single-shot fast spin echo (SSFSE) provided ‘black blood’ imaging to visualize vascular anatomy, and balanced steady-state free precession (bSSFP) produced ‘bright-blood’ contrast for assessment of cardiac structures [9, 10]. Early FCMRIs were also constrained by the fetus’s rapid heart rate and lack of a reliable in-utero gating signal, resulting in motion and temporal blurring [11]. Retrospective approaches such as self-gating and metric-optimized gating partially overcame these limitations [12]. 

Both 1.5T and 3.0T MRI systems are employed in fetal cardiac imaging today. More recently, direct beat-to-beat triggering using fetal electrocardiogram and Doppler ultrasound gating systems has enabled conventional cine and flow sequences with immediate reconstruction and temporal resolution [11–13]. Current acquisition techniques, reconstruction software, and gating strategies that enable faster, more accurate three-dimensional modeling and volume measurements allow the introduction of FCMRI into the clinical toolbox for earlier, real-time management of high-risk pregnancies, extending its potential into the mid-second trimester.

FCMRI, a less operator-dependent modality, complements FE by providing additional anatomical detail. Studies comparing FCMRI to FE have found similar sensitivity levels [14–16]. The value of FCMRI in challenging cases, marked by complex defects, poor echo views, and later gestation, lacks a clear qualitative and quantitative definition. In this meta-analysis, we aim to synthesize current evidence to estimate the DTA of FCMRI in structural malformations of the heart, great arteries, and systemic and pulmonary veins against autopsy or postnatal diagnoses, to evaluate its role in high-risk referrals, and to clarify its potential contribution to clinical decision-making and perinatal management.

## Methods

This meta-analysis was conducted in accordance with the Preferred Reporting Items for Systematic Reviews and Meta-Analyses for DTA (PRISMA-DTA) guidelines (Supporting Information S1: PRISMA checklist) [17, 18]. The protocol was registered with PROSPERO (CRD420251133115 dated September 9, 2025).

### Search strategy

A comprehensive literature search was performed in PubMed, EMBASE, Cochrane Central Register of Controlled Trials, and ClinicalTrials.gov to identify eligible studies. The search strategies are detailed in (Supporting Information S2: Table S1). Search terms included the population (fetuses), the index test (FCMRI), and the target condition (structural CTVAs). Only human studies published in English from January 1, 2000, onward were included. Both observational and experimental studies were considered. Two reviewers independently screened titles and abstracts for eligibility, followed by full-text review. The screening and study selection process was managed using Rayyan (http://rayyan.qcri.org).

### Eligibility criteria

Studies were eligible if they included fetuses undergoing cardiac MRI as an additional investigation for structural CTVAs. Confirmation was required by autopsy in cases of termination or perinatal death or by postnatal echocardiography, computed tomography, MRI, or operative findings (reference standard). Studies were excluded if they were small case series (≤ 4), focused on hemodynamic functions (e.g., blood flow, oxygen delivery), extracardiac malformations, or MRI acquisition techniques.

### Data extraction

Data extraction and methodological quality assessment were performed by three independent reviewers, and discrepancies were resolved by consensus. The CTVAs were categorized into predefined subgroups: (1) complex heart defects, (2) septal defects and valvular lesions, (3) aorta (Ao) and pulmonary artery (PA) anomalies, (4) aberrant arterial and venous structures, and (5) miscellaneous. Non-structural descriptions, such as cardiomegaly, pericardial effusion, rhabdomyomas, and cardiac malposition due to congenital diaphragmatic hernia, were excluded. A glossary of the CTVA descriptions is available in Supporting Information S2: Table S2. The complex heart defects subgroup included complex malformations that variably involved the three embryologic segments: viscero-atrial situs, ventricular loop, and truncus arteriosus. Moreover, their outcomes depend on concomitant shunts, valvular stenosis or atresia, and ventricular compliance.

The unit of analysis was the individual CTVA description, because several fetuses had more than one. The descriptions were categorized into true positive, false positive, false negative, and true negative for DTA estimation. A true positive was defined as agreement between FCMRI findings and the reference standard for a lesion, and a true negative as agreement for normal cardiac anatomy. A false negative reflected the failure of FCMRI to identify a CTVA due to a technical or interpretative error, whereas a false positive indicated a suspected lesion on FCMRI that was not substantiated by the reference standard.

In addition, gestational week at imaging, MRI protocols, and reported changes in clinical management, including modifications to delivery planning and neonatal outcomes, were tabulated. No assumptions were made about missing or unclear information.

### Assessment of quality of evidence: bias and applicability

The quality of the included studies was assessed using the Quality Assessment of Diagnostic Accuracy Studies 2 (QUADAS-2) tool. QUADAS-2 evaluates three domains for risk of bias and applicability concerns: patient selection, the index test, and the reference standard. A fourth domain, flow and timing, is assessed only for risk of bias. The QUADAS-2 domains in this study are described in Supporting Information S2: Table S3.

### Meta-analysis model and data synthesis

Several studies contained zero cells in their 2 × 2 contingency tables, precluding logit transformation. A continuity correction of 1.0 was therefore applied to all four cells (true positives and negatives; false positives and negatives), for studies with at least one zero cell in false positive only [19]. However, continuity correction was not applied to studies with zero true negative to avoid skewing specificity. This was particularly relevant as FCMRI is used as a supplemental test to FE in high-risk populations, limiting true negative representation.

A bivariate random-effects model was used to estimate pooled sensitivity and specificity, accounting for their correlation across studies [20]. Diagnostic performance was summarized using hierarchical summary receiver operating characteristic (HSROC) curves with 95% confidence and prediction ellipses to capture between-study heterogeneity [21]. Pooled estimates of sensitivity and specificity with 95% confidence intervals (CI), positive likelihood ratio, negative likelihood ratio, and diagnostic odds ratio (DOR) were reported [22]. The area of 95% prediction ellipse was also tabulated.

Analyses were conducted using Meta-DiSc 2.0, selected for its simplicity and direct implementation of these models [23–25]. Forest plots were generated using RevMan 5.4.

### Heterogeneity, publication bias, and sensitivity analysis

The hierarchical models generated for subgroup analyses by CTVAs addressed between-study heterogeneity by depicting summary points and prediction regions in HSROC space, without relying on Cochran’s Q test or Higgins’ I² statistic [21, 23]. Heterogeneity was further explored using metaregression, examining the effects of acquisition era (before 2020 vs. 2020 or later), mean gestational age (< 30 vs. ≥30 weeks), MRI field strength (1.5T vs. 3T), and the use of gating techniques (present vs. absent). A *p*-value < 0.05 was considered statistically significant.

Formal assessment of publication bias was not performed due to the small number of included studies (< 10) and the known limitations of funnel plot asymmetry tests based on DOR in DTA meta-analyses. Although a Deeks funnel plot was not feasible, graphical inspection of study-specific DORs was undertaken. Sensitivity analyses were carried out in R using the *mada* package, applying a 0.5 continuity correction to ensure model convergence.

## Results

### Study selection and study characteristics

Of the 2,098 studies identified, 47 underwent full-text review, and 18 were assessed for eligibility. Five conference abstracts and two studies with partial data were excluded after full-text review [26–32]. Two studies with overlapping datasets and two studies with no normal fetal cardiac imaging (true negative) were excluded after data extraction [14, 16, 33, 34]. 

**Fig. 1:**
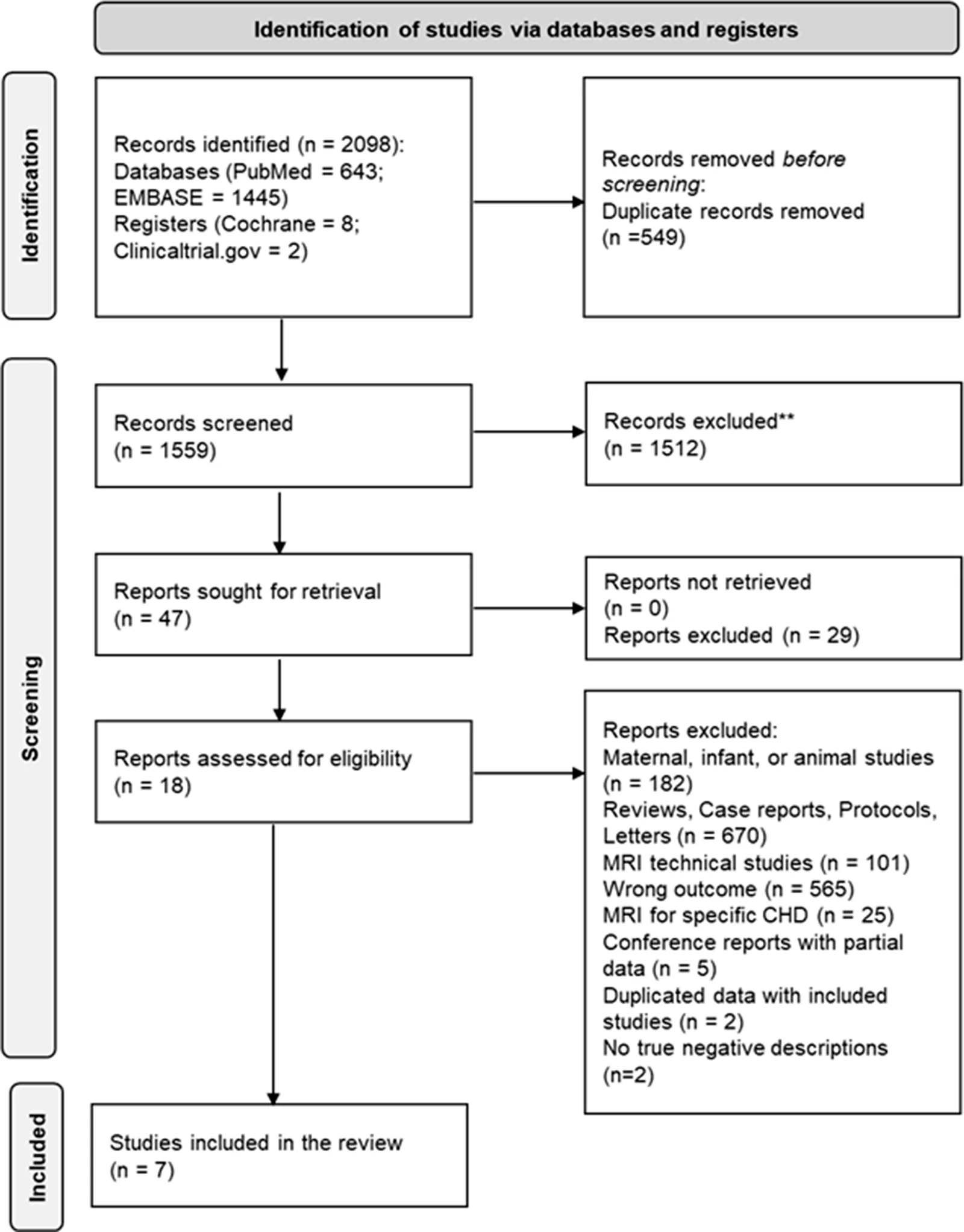
Flowchart summarizing inclusion of studies in the review

Figure 1 shows the PRISMA 2020 study selection flow diagram. Thus, seven observational studies were included in the meta-analysis. Of the 2,182 lesion-level diagnostic descriptions, 449 CTVAs were identified. The median gestational age at MRI was 31 weeks. Study characteristics are detailed in Supporting Information S3: Table S4, and the FCMRI workflow in Supporting Information S3: Table S5.

### Risk of bias using QUADAS-2

**Fig. 2:**
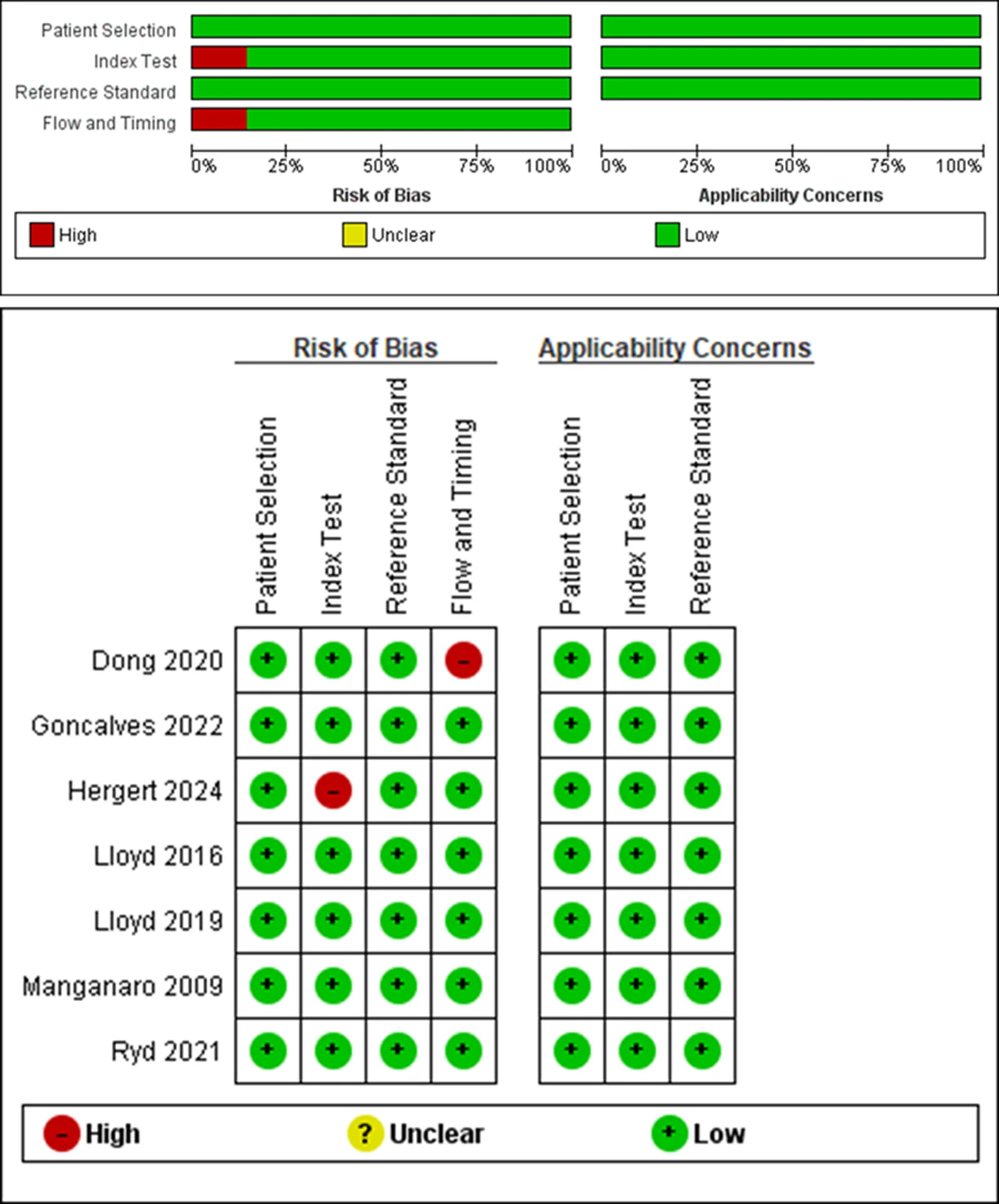
Risk of bias and applicability concerns assessment using QUADAS-2. The top panel shows the judgments about each domain presented as percentages across the included studies. The bottom panel shows judgments about each domain for each included study

Figure 2 presents the QUADAS-2 risk-of-bias summary and graphical overview. Hergert et al. [35] reported limited radiologist experience, while Dong et al. [36] raised concerns in the flow and timing domain due to second-trimester MRI acquisition.

### Qualitative and quantitative data of individual studies

**Fig. 3:**
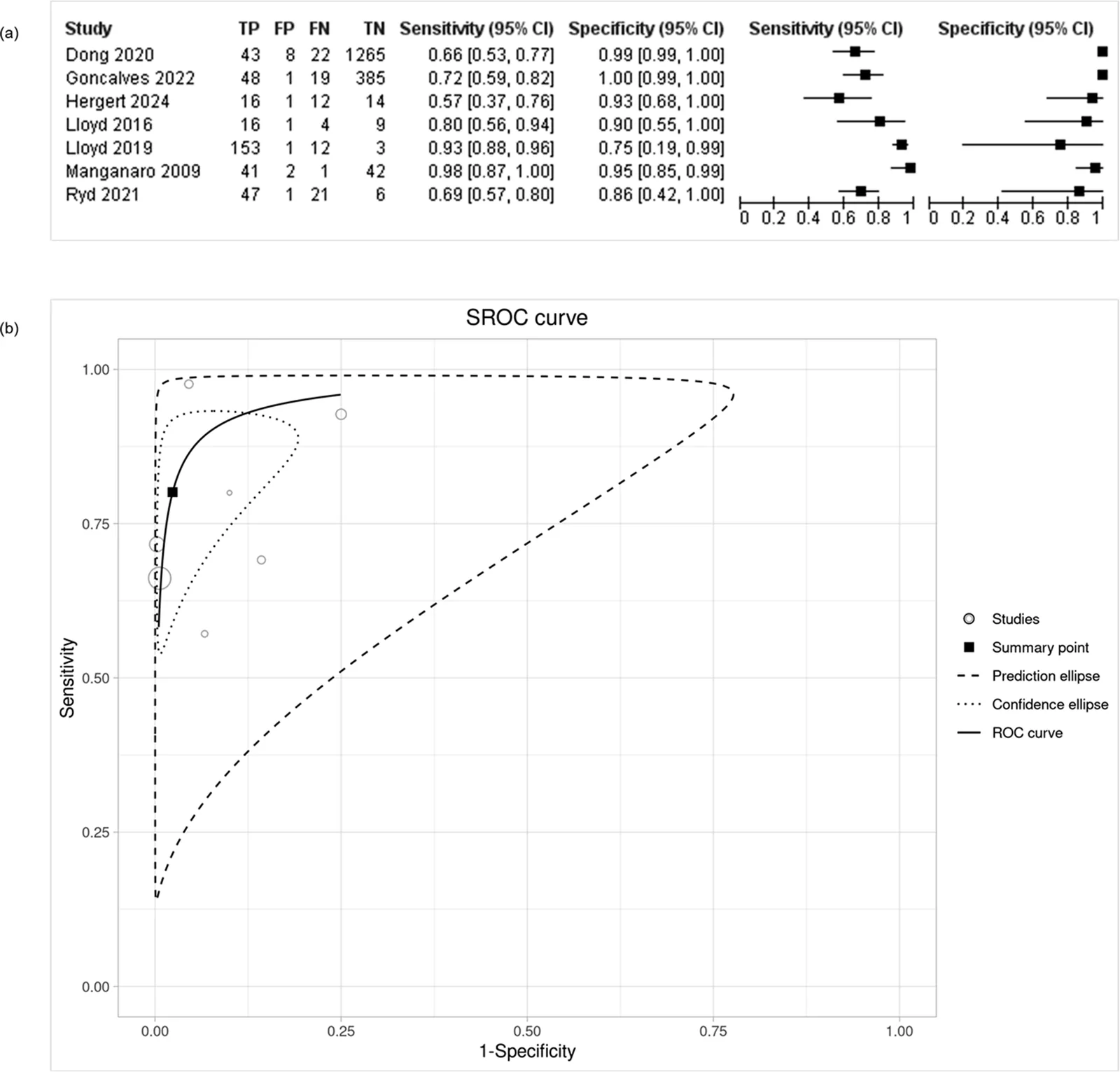
Summary statistics of pooled fetal cardiac MRI descriptions in the diagnosis of cardiac and thoracic vascular anomalies: (**a**) forest plots, and (**b**) hierarchical summary receiver operating characteristic curves

Table 1 presents the diagnostic classifications across five structural categories. Dong et al. [36] (*n* = 1,337) and Goncalves et al. [15] (*n* = 415) contributed the greatest statistical weight, particularly to pooled specificity estimates (Fig. 3a). Both reported diagnostic challenges in complex heart defects, septal and valvular lesions, and aortic arch (AA) anomalies, reflected in comparatively lower sensitivity. Hergert et al. [35] (*n* = 28) demonstrated reduced sensitivity due to missed AA anomalies, septal, and valvular lesions.

**Table 1:** Diagnostic performance of fetal cardiac magnetic resonance imaging for cardiac and thoracic vessel anomalies compared with postnatal diagnosis or autopsy

Author, Year	Category	True Positive	*n*	False Positive	*n*	False Negative	*n*	True Negative	*n*	Excluded descriptions
Dong 2020 [36] (*n* = 1,337)	Complex	Criss-cross heart (1), AVCD (3), TGA (5), APVC (2), TOF (4), DORV (3), VSD with pulmonary atresia (1), HLHS (3), Ebstein (1)	23		0	DORV (1), VSD with pulmonary atresia (1), TAPVC (3)	5		0	Unclosed ductus arteriosus (6), pericardial effusion (2), anomalous left coronary artery (1)
	Septal defects and valvular	VSD (3), PS (1)	4	VSD (5)	5	VSD (6), ASD (5), PS (2)	13		0	
	Aorta & PA	CoA (2), interrupted AA (1), RAA (5), DAA (1)	9	CoA (3)	3	CoA (4)	4		0	
	Aberrant vessels	LSVC (5)	5		0		0		0	
	Miscellaneous	Heterotaxia (2)	2		0		0	Normal (1265)	1265	
	Total descriptions		43		8		22		1265	
Goncalves 2022 [15] (*n* = 415)	Complex	Common atrium (1), right atrial isomerism (2), TGA (2), single ventricle (2), AVCD (1), Tricuspid atresia (1), TA (1), DORV (2), TOF with pulmonary atresia (2), TAPVC (3), PAPVC (1), Ebstein anomaly (2), HLHS (6)	26	PAPVC (1)	1	AVCD unbalanced (2), DORV (3), TGA (1), TOF with pulmonary atresia (1)	7		0	Cardiomegaly (3), pericardial lesions (2), hydrops (1), cardiomyopathy (1)
	Septal defects and valvular	VSD (3), right ventricle disproportion (1), MV atresia (2)	6		0	TV dysplasia with regurgitation (1)	1	No VSD (1)	1	
	Aorta & PA	Hypoplastic AA (1), RAA (1), pulmonary atresia (4), Hypoplastic PA (2)	8		0	CoA (1), hypoplastic AA (1), APCs (2)	4		0	
	Aberrant vessels	scimitar vein (1), LSVC (2), interrupted IVC (1), midline IVC (1), persistent right umbilical vein (1)	6		0	Aberrant right SCA (2), LSVC (3), retro-aortic BC vein (1)	6		0	
	Miscellaneous	Dextrocardia (2)	2		0	Poor image (1)	1	Normal (384)	384	
	Total descriptions		48		1		19		385	
Hergert 2024 [35] (*n* = 28)	Complex	AVCD (2), TOF (2), Ebstein anomaly (1)	5		0		0		0	Dextrocardia in CDH
	Septal defects and valvular	ASD (3), VSD (1), BAV with AS (1), PS (1), absent PV (1), MV dysplasia (1)	4		0	BAV with AS (1), ASD (1), VSD (2)	5	No VSD (2)	2	
	Aorta & PA	CoA (2), hypoplastic AA (2), narrow aortic isthmus (1), hypoplastic PA (1)	6		0	RAA (1), interrupted AA (1), hypoplastic AA (1), APCs (1), pulmonary atresia (1)	5		0	
	Aberrant vessels		0		0		0		0	
	Miscellaneous		0		0	Poor image in control (1)	1	Normal controls (11)	11	
	Total descriptions		15		0		11		13	
Lloyd 2016 [37] (*n* = 22)	Complex	HLHS (1), HLHS with RAS (1), TA (1)	3		0		0		0	Dextrocardia in diaphragmatic hernia (1), patent ductus (1), no postnatal diagnosis (2), rhabdomyomas (2)
	Septal defects and valvular	VSD (1), PV dysplasia (1), right ventricle disproportion (2)	4		0		0		0	
	Aorta & P	CoA (3), interrupted AA (1), RAA (1), pulmonary vein anomaly (1)	6		0	CoA (1), RAA (1)	2	Isolated arterial duct tortuosity (1), no pulmonary vein anomaly (2), no CoA (1)	4	
	Aberrant vessels	Aberrant LSCA (1) from a RAA, LSVC (1)	2		0	Aberrant left SCA (1) from a RAA	1			
	Miscellaneous		0		0			Normal twin (2), no structural malformations (2)	4	
	Total descriptions		15		0		3		8	
Lloyd 2019 [40] (*n* = 82)	Complex	TOF (6), DORV (3), VSD with pulmonary atresia (4), TGA (5), TA (1), PAPVC (1), TAPVC (1), AV-VA discordance (1), HLHS (6)	27		0	PAPVC (1)	1		0	Extracardiac malformation (1), no postnatal diagnosis (2)
	Septal defects and valvular	VSD (5), absent PV (2), MS (1), AS (1)	9		0		0		0	
	Aorta & PA	CoA (16), RAA (26), DAA with one hypoplastic (3) dilated AA (1); hypoplastic AA (26), hypoplastic ascending and AA (4), aortic aplasia (1), APCs (5), PA hypoplasia (4), dilated PA (2)	88		0	DAA with hypoplastic left arch as RAA (2)	2	No CoA (2)	2	
	Aberrant vessels	Aberrant SCA (21), common origin of BC and left common carotid artery (1), bilateral or LSVC (4), interrupted IVC (1)	27		0	Aberrant left SCA in RAA (4), retro-aortic BC vein (3), bilateral SVC (1)	8		0	
	Miscellaneous	Dextrocardia (1)	1		0		0		0	
	Total descriptions		152		0		11		2	
Manganaro 2009 [34] (*n* = 82)	Complex	Right atrial isomerism (1), Trilocular heart (1), TOF (6), AVCD (2), TGA (2), TA (1), Tricuspid atresia (1), HLHS (5)	19		0		0	Normal in echo-diagnosed HLHS (1)	1	Cardiomegaly (10), pericardial effusion (4), rhabdomyomas (3), pericardial lymphangioma (1), cardiomyopathy (1)
	Septal defects and valvular	VSD (4), ASD (2), Valvular: PS (2), Mitral-Aortic (1)	9	VSD (2)	2	VSD in TA (1)	1		0	
	Aorta & PA	CoA (1), RAA (3), hypoplastic AA (1), PA hypoplasia (1)	6		0		0		0	
	Aberrant vessels		0		0		0		0	
	Miscellaneous	Malrotations (7)	7		0		0	Controls (40), normal twin (1)	41	
	Total descriptions		41		2		1		42	
Ryd 2021 [39] (*n* = 31)	Complex	AVCD unbalanced (3), AVCD complete (1), TGA (3), DORV (1), DILV (1), HLHS (3), HLHS with RAS (1)	13		0	TAPVR (1)	1		0	
	Septal defects and valvular	VSD (5), ASD (2), AS (1), PS (1), MV dysplasia (5), hypoplastic AV (1), hypoplastic left ventricle (2), bipartite right ventricle (1)	18		0	VSD (1), BAV (2), sub-aortic area not visualized (1), aortic valve hypoplasia (1), MV annulus not visualized (3)	8	No AS	1	
	Aorta & PA	CoA (4), AA hypoplasia (7), pulmonary atresia (1), pulmonary atresia with IVS (1)	11	CoA (1)	1	CoA (5), AA not visualized (3), DAA with hypoplastic left as RAA (1), pulmonary atresia (1), pulmonary branch arteries not visualized (1)	11	No CoA (4)	4	
	Aberrant vessels	LSVC (1), interrupted IVC (2)	3		0		0		0	
	Miscellaneous	Dextrocardia (2)	2		0	Poor image quality (1)	1	Normal (echo not diagnostic)	1	
	Total descriptions		47		1		21		6	

AA- aortic arch, APC-aortopulmonary collaterals, APVC-anomalous pulmonary venous connection, AS-aortic stenosis, ASD-atrial septal defect, AVCD-atrioventricular canal defect, BAV-bicuspid aortic valve, BC-brachiocephalic, CoA-coarctation of aorta, DAA-double aortic arch, DILV-double inlet left ventricle, DORV-double outlet right ventricle, HLHS-hypoplastic left heart syndrome, IAS-intact atrial septum, IVC-inferior vena cava, IVS-intact ventricular septum, LSVC-left superior vena cava, MS-mitral stenosis, MV-mitral valve, PA-pulmonary artery, PAVC-partial anomalous pulmonary venous connection, PS-pulmonary stenosis, PV-pulmonary valve, RAA-right aortic arch, RAS-restrictive atrial septum, SCA-subclavian artery, TAPVC-total anomalous pulmonary venous connection, TGA-transposition of the great arteries, TOF-tetralogy of Fallot, TV-tricuspid valve, VA-ventriculo arterial, VSD-ventricular septal defect

Lloyd et al. [37] (2016; *n* = 22) showed strong performance for complex heart defects and AA anomalies. Their 2019 cohort (*n* = 82) demonstrated particularly robust data for thoracic vascular morphology with 117 true descriptions of AA anomalies and aberrant vessels. Manganaro et al. [38] (*n* = 82) demonstrated high overall diagnostic accuracy with few false positive. Ryd et al. [39] (*n* = 31) exhibited lesion-dependent variability, with high false negative rates for septal defects and AA anomalies, reducing sensitivity.

### Quantitative synthesis of included studies: summary statistics

In bivariate analysis, pooled sensitivity for detecting structural cardiac malformations was 0.80 (95% CI: 0.66–0.89), and pooled specificity was 0.98 (95% CI: 0.92–0.99), indicating high overall diagnostic performance (Fig. 3a). The HSROC curve demonstrated a 95% prediction ellipse area of 0.29, reflecting moderate dispersion in study-level accuracy (Fig. 3b). The pooled positive likelihood ratio indicated strong rule-in capability, while the negative likelihood ratio suggested moderate rule-out performance. The DOR showed moderate discriminatory power. Continuity corrections for zero cells increased within-study variance, yielding wider confidence intervals for the likelihood ratios and DOR (Table 2).

**Table 2:** Summary statistics of diagnostic accuracy of fetal cardiac MRI in structural cardiac and thoracic vascular anomalies and subgroups

Data	Sensitivity (95% CI)	Specificity (95% CI)	Correlation	Positive likelihood ratio (95% CI)	Negative likelihood ratio (95% CI)	Diagnostic odds ratio (95% CI)	Area of 95% prediction ellipse
Pooled cardiac and thoracic vessel anomalies	0.80 (0.66, 0.89)	0.98 (0.92, 0.99)	-0.59	33.85 (9.82, 116.74)	0.20 (0.12, 0.36)	165.87 (48.39, 568.57)	0.29
**Sub-groups**							
Complex heart defects	0.88 (0.79, 0.93)	0.99 (0.97, 1.00)	-1.0	107.01 (31.86, 359.49)	0.13 (0.07, 0.21)	856.67 (256.37, 2778.43.30)	0.04
Septal defects and valvular lesions	0.70 (0.47, 0.86)	0.99 (0.97, 1.00)	-0.69	63.58 (22.57, 179.09)	0.31 (0.16, 0.59)	207.98 (60.85, 710.82)	0.09
Aorta and pulmonary artery lesions	0.75 (0.56, 0.88)	0.99 (0.95, 1.00)	-0.41	55.71 (15.16, 204.76)	0.25 (0.13, 0.49)	221.02 (53.54, 912.49)	0.39
Aberrant vessels	0.69 (0.58, 0.79)	0.99 (0.98, 1.00)	-	98.57 (29.03, 334.73)	0.31 (0.22, 0.44)	320.31 (85.53, 1199.60)	-

### Quantitative synthesis of included studies: subgroup analysis

Subgroup analysis by CTVA showed best diagnostic performance for complex heart defects, with high sensitivity [0.88 (95% CI: 0.79–0.93)], high specificity [0.99 (95% CI: 0.97–1.00)], and a tightly clustered SROC 95% prediction ellipse with an area of 0.04, indicating greater inter-study consistency (Table 2, Supporting Information S3: Fig. S1). For intracardiac septal and valvular lesions, pooled specificity remained high at 0.99 (95% CI: 0.97–1.00), whereas sensitivity was moderate at 0.70 (95% CI: 0.47–0.86), suggesting persistent diagnostic limitations in subtle septal and valvular pathology. The prediction ellipse area was 0.09 (Table 2, Supporting Information S3: Fig. S2).

Similarly, for Ao and PA anomalies, specificity was high at 0.99 (95% CI: 0.95–1.00), with moderate sensitivity of 0.75 (95% CI: 0.56–0.88). The wider prediction ellipse (0.39) indicated greater variability in detecting AA variants (Table 2, Supporting Information S3: Fig. S3). For aberrant vessels, pooled sensitivity was lower at 0.69 (95% CI: 0.58–0.79), while specificity remained high at 0.99 (95% CI: 0.98–1.00). As only three studies contributed data, the corresponding SROC curve could not be generated (Table 2, Supporting Information S3: Fig. S4).

The metaregression results for the effects of acquisition era, gestational age, MRI field strength, and cardiac gating are shown in Table 3. Studies conducted before 2020 demonstrated significantly higher sensitivity than those published in 2020 or later [relative sensitivity 1.37 (95% CI 1.24–1.51); *p* < 0.001]. Gating technique and mean gestational age were not significantly associated with either sensitivity or specificity. The effect of MRI field strength could not be reliably evaluated.

**Table 3:** Metaregression of included studies grouped by acquisition era, gestational age, MRI field strength, and cardiac gating

Subgroups	Sensitivity (95% CI)	Specificity (95% CI)	Metaregression			
			Relative Sensitivity (95% CI)	*p*-value	Relative Specificity (95% CI)	*p*-value
Study year						
Before 2020 [34, 37, 40]	0.93 (0.88, 0.95)	0.93 (0.83, 0.97)	1.37 (1.24, 1.51)	< 0.001	0.93 (0.87, 1.00)	0.026
2020 & later [15, 35, 36, 39]	0.68 (0.61, 0.73)	0.99 (0.99, 1.00)	Reference group		Reference group	
Mean gestational age						
< 30 weeks [34, 36]	0.86 (0.60, 0.96)	0.99 (0.90, 1.00)	1.11 (0.85, 1.46)	0.465	1.02 (0.96, 1.09)	0.504
≥ 30 weeks [15, 35, 37, 39, 40]	0.78 (0.60, 0.89)	0.96 (0.85, 0.99)	Reference group		Reference group	
Magnetic field strength^a^			Not estimable		Not estimable	
1.5 Tesla [34, 36, 37, 39, 40]	0.84 (0.71, 0.92)	0.98 (0.93, 0.99)	-	-	-	-
3 Tesla [35]	0.57 (0.20, 0.88)	0.94 (0.50, 1.00)	-	-	-	-
Cardiac Gating						
Nongated [34, 36, 37, 40]	0.87 (0.76, 0.94)	0.97 (0.86, 0.99)	1.30 (0.98–1.72)	0.053	0.98 (0.92, 1.04)	0.526
Gated [15, 35, 39]	0.67 (0.48, 0.82)	0.99 (0.89, 1.00)	Reference group		Reference group	

^a^metaregression was not feasible with only one study in the 3T group, and Goncalves et al. was excluded as both 1.5T and 3T were used

### Perinatal management and clinical decision-making

Only three studies evaluated the impact of FCMRI on clinical decision-making where FE was either not feasible or yielded uncertain findings [15, 36, 39]. Goncalves et al. [15] reported that MRI-detected extracardiac anomalies altered prognosis and postnatal management in 32% of fetuses. Ryd et al. [39] found that MRI contributed to improved decision-making in 84% of fetuses across several contexts, including AA morphology in CoA, determination of uni- versus biventricular outcomes in complex heart defects, and identification of a nutmeg lung pattern suggestive of pulmonary lymphangiectasia in hypoplastic left heart syndrome (HLHS). The delineation of additional features changed prenatal prognostication and counseling, delivery hospital selection, and prostaglandin decision. In contrast, Dong et al. [36] reported no significant impact of MRI on clinical decision-making. Collectively, these findings indicate that the clinical utility of FCMRI in perinatal management is context-dependent and varies by specific clinical scenario.

### Heterogeneity and sensitivity analysis

The HSROC curves demonstrated between-study heterogeneity. Variability was attributable to differences in MRI field strength and acquisition protocols, sample size, gestational week at imaging, lesion spectrum, and radiologist experience. Studies by Hassan et al. [33], Hergert et al. [35], Ryd et al. [39], and Vollbrecht et al. [16] contributed most substantially to between-study variability. In sensitivity analysis, application of a 0.5 continuity correction yielded pooled estimates comparable to those obtained using a 1.0 correction (Supporting Information S4: Sensitivity analysis).

## Discussion

This meta-analysis provides a comprehensive synthesis of evidence on the performance of FCMRI in diagnosing CTVAs. A pooled bivariate analysis of seven studies demonstrated high sensitivity and specificity, confirming strong overall diagnostic accuracy. Although the 95% prediction ellipse indicated moderate between-study dispersion, overall test performance remained consistently robust. In our subgroup analysis, the highest DTA was observed in the complex heart defects subgroup. Sensitivity was lower for isolated AA and PA lesions. For the smaller aberrant vascular subgroup, the limited number of cases precluded reliable HSROC modeling, warranting cautious interpretation. Clinical utility of FCMRI in decision-making varies by specific CTVA.

### FCMRI as an adjunct imaging modality

Our findings support FCMRI as a clinically meaningful adjunct to FE, particularly in situations where acoustic windows constrain echocardiographic assessment or when third-trimester evaluation is critical for perinatal management. Although FE remains the primary imaging modality, its diagnostic performance for the 4CV, OTVs, and 3TV protocol declines in the third trimester, with pooled sensitivity decreasing from 0.89 (95% CI: 0.82–0.95) in the second trimester to 0.81 (95% CI: 0.75–0.86) in the third trimester [6]. Importantly, FCMRI aided in assessing septal patency, ventricular adequacy, atrioventricular valve annular size, pulmonary lymphangiectasia, and associated extracardiac anomalies, which had direct implications for prognosis and perinatal management [39]. 

Our subgroup analysis by CTVAs suggests that visualization is superior for anatomical structures assessed on four-chamber views, whereas evaluation of the AA and aberrant vascular anomalies remains challenging. Earlier non-gated studies, such as Manganaro et al. [38], reported substantial limitations in visualizing the AA, PA, and the interatrial septum. In contrast, Lloyd et al. [37, 40] demonstrated the value of FCMRI for delineating thoracic vascular anatomy. The authors identified three diagnostic categories in which MRI provided incremental benefit: AA hypoplasia and CoA; right AA/double AA; and pulmonary vascular abnormalities.

Our study also demonstrated between-study heterogeneity, attributable primarily to the CTVA type and image acquisition era. Similar heterogeneity has been reported in a meta-analysis of FE, where pooled sensitivity was high at 0.90 (95% CI: 0.86–0.93) for the 4CV, OTVs, and 3TV protocol, but individual study estimates ranged from 0.68 to 1.00 [41]. Likewise, the pooled specificity approached 1.00, mirroring our findings and likely reflecting the predominance of high-risk pregnancies referred after abnormal obstetric ultrasonography. Furthermore, several studies included in the present review also reported good concordance between FE and FCMRI [15, 35, 37]. 

Our metaregression findings on higher sensitivity in studies conducted before 2020 should be interpreted cautiously given the small number of studies. The results likely reflect differences in case selection, methodologies, and more conservative diagnostic approaches, potentially reducing false-positive diagnoses and increasing specificity at the expense of sensitivity.

### FCMRI in specific anomalies

Although there is currently no practice guideline for the clinical role of FCMRI, focused case series support FCMRI’s role in specific CTVAs. Serrano et al. [42] highlighted the role of FCMRI in optimizing delivery-site planning for prenatally diagnosed HLHS. Li et al. [43] reported superior accuracy of FCMRI over FE for AA anomalies, attributed to improved visualization of the transverse arch. Efforts to enhance sensitivity for detecting aortic lesions increasingly integrate structural arch morphology, quantitative flow assessment, and three-dimensional reconstruction into MRI workflows to improve the prediction of postnatal obstruction [44–46]. Studies on right AA, double AA variants and persistent left superior vena cava further demonstrate FCMRI’s value in clarifying vascular anatomy when FE is limited [43, 47, 48]. 

### Strengths and Limitations

To our knowledge, this is the first DTA meta-analyses synthesizing lesion-level performance of FCMRI for structural CTVAs using postnatal confirmation as the reference standard. The application of a bivariate random-effects model enabled joint estimation of sensitivity and specificity, and lesion-specific subgroup analyses enhanced clinical interpretability. Inclusion of prediction regions further enhances translational relevance by reflecting expected performance across diverse clinical settings.

Interpretation should consider several limitations. Although hierarchical DTA methods can be applied to small datasets, the inclusion of only seven studies may have limited statistical power, hindered adequate exploration of publication bias, and led to potential overinterpretation of subgroup analyses. Because the unit of analysis was the individual lesion rather than the fetus, multiple observations from the same fetus may have introduced clustering effects and violated assumptions of statistical independence. Clinical and methodological heterogeneity, including variation in CTVA descriptions and subgroup distributions, could not be fully mitigated, particularly for thoracic vascular anomalies.

Low false positive counts across several studies necessitated continuity corrections, potentially widening CIs and limiting precision despite random-effects modeling. Although two studies were excluded because they contained no true-negative cases [13, 33], several included studies still underrepresented true negatives, introducing spectrum bias [16, 19, 39]. The specificity estimates may not be generalizable to broader populations. In addition, these studies were conducted in tertiary centers with overlapping teams interpreting both FE and FCMRI and could have influenced MRI interpretation.

Our study showed between-study heterogeneity, attributable to differences in MRI generation, gating strategies, radiologist expertise, and lesion spectrum. Technological advances, particularly Doppler ultrasound gating and optimized sequence acquisition, have since mitigated some of these limitations. Nevertheless, FCMRI remains highly technical, restricting its availability primarily to advanced gestations and tertiary referral centers.

## Conclusion

Fetal cardiac MRI demonstrated high specificity and moderate sensitivity in evaluating structural CTVAs. Available evidence suggests that FCMRI can provide complementary anatomical information to FE, particularly in late gestation and selected high-risk pregnancies with inconclusive echocardiographic findings or limited acoustic windows. Its diagnostic performance varied across lesion subtypes, with aortic and aberrant vascular anomalies remaining particularly challenging to diagnose prenatally. However, the current evidence is limited by the small number of studies, methodological heterogeneity, and the underrepresentation of true-negative cases. Larger prospective studies on FCMRI are needed to better define the incremental diagnostic value, clinical utility, and role in perinatal management.

## Supplementary Information

Below is the link to the electronic supplementary material.

Supplementary Material 1

Supplementary Material 2

Supplementary Material 3

Supplementary Material 4

## Data Availability

All data generated or analyzed are included in the manuscript and supplementary files.

## Abbreviations

3VT: Three-vessel trachea view
4CV: Four-chamber view
AA: Aortic arch
APC: Aortopulmonary collaterals
APVC: Anomalous pulmonary venous connection
AS: Aortic stenosis
ASD: Atrial septal defect
AVCD: Atrioventricular canal defect
BAV: Bicuspid aortic valve
BC: Brachiocephalic
CoA: Coarctation of aorta
CHD: Congenital heart disease
CI: Confidence interval
CTVA: Cardiac and thoracic vascular anomalies
DAA: Double aortic arch
DILV: Double inlet left ventricle
DOR: Diagnostic odds ratio
DORV: Double outlet right ventricle
DTA: Diagnostic test accuracy
FCMRI: Fetal cardiac magnetic resonance imaging
FE: Fetal echocardiography
HLHS: Hypoplastic left heart syndrome
HSROC: Hierarchical summary receiver operating characteristic
IAS: Intact atrial septum
IVC: Inferior vena cava
IVS: Intact ventricular septum
LSVC: Left superior vena cava
MS: Mitral stenosis
MV: Mitral valve
OTV: Outflow tract view
PA: Pulmonary artery
PAVC: Partial anomalous pulmonary venous connection
PS: Pulmonary stenosis
PV: Pulmonary valve
RAA: Right aortic arch
RAS: Restrictive atrial septum
SCA: Subclavian artery
TAPVC: Total anomalous pulmonary venous connection
TGA: Transposition of the great arteries
TOF: Tetralogy of Fallot
TV: Tricuspid valve
VA: Ventriculo arterial
VSD: Ventricular septal defect
