# Supplementary material for: Diagnostic accuracy of fetal cardiac magnetic resonance imaging in cardiac and thoracic vascular anomalies: a meta-analysis
Source: BMC Med Imaging. 2026 Jun 23;26:530. doi: 10.1186/s12880-026-02521-x (PMC13681847; doi:10.1186/s12880-026-02521-x)
Supplement: Supplementary file 2 [file 12880_2026_2521_MOESM2_ESM.docx]

**Supporting Information S2.**

Additional information related to the Methods section, namely, Tables S1-S3.

**Table S1. Database search strategy for the meta-analysis on diagnostic test accuracy of fetal cardiac MRI in congenital heart diseases.**

| **Search Engine** | **Search strategy** | **Date** | **Abstracts retrieved** |
| --- | --- | --- | --- |
| PubMed | (("fetus"[Title/Abstract] OR fetal"[Title/Abstract] OR "foetus"[Title/Abstract] OR "foetal"[Title/Abstract] OR "prenatal"[Title/Abstract] OR "antenatal"[Title/Abstract]) AND ("humans"[MeSH Terms] AND 2000/01/01:3000/12/12[Date - Publication] AND "english"[Language]) AND (("cardia"[Title/Abstract] OR "cardiovascular"[Title/Abstract] OR "heart"[Title/Abstract] OR "aorta"[Title/Abstract] OR "pulmonary artery"[Title/Abstract]) AND ("humans"[MeSH Terms] AND 2000/01/01:3000/12/12[Date - Publication] AND "english"[Language])) AND (("magnetic resonance"[Title/Abstract] OR "MRI"[Title/Abstract] OR "mr imaging"[Title/Abstract] OR "CMRI"[Title/Abstract]) AND ("humans"[MeSH Terms] AND 2000/01/01:3000/12/12[Date - Publication] AND "english"[Language]))) AND ((humans[Filter]) AND (2000:3000/12/12[pdat]) AND (english[Filter])) | 15 September, 2025 | 643 |
| EMBASE | ('fetus':ti,ab,kw OR 'fetal':ti,ab,kw OR 'foetus':ti,ab,kw OR 'foetal':ti,ab,kw OR 'prenatal':ti,ab,kw OR 'antenatal':ti,ab,kw) AND ('cardia':ti,ab,kw OR 'cardiovascular':ti,ab,kw OR 'heart':ti,ab,kw OR 'aorta':ti,ab,kw OR 'pulmonary artery':ti,ab,kw) AND ('magnetic resonance':ti,ab,kw OR 'mri':ti,ab,kw OR 'mr imaging':ti,ab,kw OR 'cmri':ti,ab,kw) AND ('humans'/exp OR 'humans') AND [2000 to 3000-12-12]/pd AND ('english'/exp OR 'english’) | 15 September, 2025 | 1455 |

**Table S2. A glossary of cardiac and vascular anomalies described in the meta-analysis.**

| **Category** | **Anomalies** |
| --- | --- |
| **Complex congenital heart defects** | complete atrioventricular septal defect, corrected transposition of the great arteries, corrected transposition of the great arteries with tricuspid valve atresia, double inlet left ventricle, double outlet right ventricle, Ebstein anomaly of the tricuspid valve, hypoplastic left heart syndrome with intact atrial septum/ restrictive atrial septum, mitral valve atresia, partial anomalous pulmonary venous connection, pulmonary atresia – isolated or with ventricular septal defect, right atrial isomerism, right ventricular hypoplasia associated with dextro-transposition of the great arteries with double outlet right ventricle, single ventricle physiology, tetralogy of Fallot with or without pulmonary atresia, tri-locular or three-chambered heart, total anomalous pulmonary venous connection, tricuspid valve atresia, truncus arteriosus, unbalanced or balanced atrioventricular septal defect |
| **Septal and valvular lesions** | absent pulmonary valve, aortic stenosis, atrial septal defect, bicuspid aortic valve, bipartite right ventricle, combined mitral and aortic valve lesion, hypoplastic aortic valve, hypoplastic atrioventricular valve, hypoplastic left ventricle, mitral stenosis, mitral valve atresia/dysplasia, pulmonary stenosis, pulmonary valve dysplasia, right ventricular disproportion, right ventricular diverticulum, thickened tricuspid valve, tricuspid valve dysplasia, valvular pulmonary stenosis, ventricular septal defect |
| **Aorta and pulmonary arterial lesions** | coarctation of the aorta, absence or failure of development of part of the aorta, anomalous pulmonary venous anatomy, aortopulmonary collaterals, dilated aortic arch, dilated pulmonary artery, double aortic arch isolated or with hypoplastic left arch and dominant right aortic arch, hypoplastic aortic arch, hypoplastic ascending aorta and aortic arch, interrupted aortic arch, isolated pulmonary atresia, narrowing of the aortic isthmus, pulmonary artery hypoplasia, right aortic arch |
| **Aberrant arteries and veins** | aberrant left subclavian artery arising from a right aortic arch, bilateral superior vena cava or left superior vena cava, brachiocephalic vein passing behind the aorta, common origin of the brachiocephalic artery and left common carotid artery, inferior vena cava – interrupted/midline, persistent left superior vena cava, persistent right umbilical vein |
| **Miscellaneous** | dextrocardia, heterotaxy, malrotations |

**Table S3. QUADAS-2 risk of bias and applicability criteria.**

| **Domain** | **Low risk of bias** | **Low concern for applicability** |
| --- | --- | --- |
| **Domain 1. Patient selection**  Fetuses evaluated for cardiovascular anomalies | - Consecutive or random sampling where fetuses are followed up until birth; avoids inappropriate exclusion criteria | - Included patients had prior testing with fetal echocardiogram, defined intended use of index test (better structure delineation), setting (tertiary radiology center/ fetal medicine unit) |
| **Domain 2. Index test**  Fetal cardiac MRI | - Pre-specified threshold: MRI Tesla, technique, standardized protocol or reported by radiologists competent in fetal imaging, and not experimental - Interpretation was without the knowledge of the postnatal diagnosis | - The MRI technique, acquisition, and interpretation were consistent with structural malformation assessment and the image was conducted in a clinical setting |
| **Domain 3. Reference standards** Postnatal diagnosis | - The reference standards, namely the imaging modality or autopsy, were appropriate for correctly identifying structural malformations, and the investigators were blinded to the MRI results | - The postnatal imaging techniques or autopsy appropriately described the structural malformation |
| **Domain 4. Patient flow and timing** | - The interval between the index test and the reference standard was appropriate - The article describes patients who did not receive the index test or reference standards | Not applicable |
