# Supplementary material for: Diagnostic accuracy of fetal cardiac magnetic resonance imaging in cardiac and thoracic vascular anomalies: a meta-analysis
Source: BMC Med Imaging. 2026 Jun 23;26:530. doi: 10.1186/s12880-026-02521-x (PMC13681847; doi:10.1186/s12880-026-02521-x)
Supplement: Supplementary file 3 [file 12880_2026_2521_MOESM3_ESM.docx]

**Supporting Information S3**

Additional information related to the Results section, namely, Tables S4-S5, and Figures S1-S4.

**Table S4. Characteristics of studies and patient selection included in the meta-analysis.**

| **Author, Year** | **Design & data type** | **Objectives** | **Sample size** | **Inclusion criteria** | **Exclusion criteria** | **Mean Gestation week** |
| --- | --- | --- | --- | --- | --- | --- |
| Dong 2020^36^ | Observational:  Jan 2005 to Oct 2019 | Utility of fetal CMRI to evaluate CHD | 1619  (71 with confirmed CHD) | Referred for fetal MRI due to technical limitations of echo | # no postnatal diagnosis (240)  # other HD (33) | 24.5 |
| Goncalves 2022^15^ | Observational:  Jan 2015 to Jun 2019 | Utility of fetal MRI to evaluate cardiac and extracardiac malformations in fetuses with suspected CHD in echocardiogram | 424 (40 with suspected CHD) | Fetal CHD in echocardiogram with or without extracardiac anomalies in obstetric ultrasound | # maternal claustrophobia (1) # no fetal echo (3)  # no postnatal diagnosis (4) | 31.4 |
| Hergert 2024^35^ | Observational: published in 2024 | Diagnostic performance of fetal CMRI using DUS gating | 29  (17 with CHD and 12 without) | Fetus with and without CHD in echo | - | 34.4 |
| Lloyd 2016^37^ | Observational:  Jun 2014 to May 2015 | Diagnostic benefit of fetal MRI for additional information over echo | 22 | Fetal CHD in echo, referred for additional imaging | - | 31 |

Table S4. Continued…

| **Author, Year** | **Study design and data collection period** | **Objectives** | **Sample size** | **Inclusion criteria** | **Exclusion criteria** | **Mean Gestation week** |
| --- | --- | --- | --- | --- | --- | --- |
| Lloyd 2019^40^ | Observational:  Oct 2015 to Jun 2017 | Utility of motion-corrected 3D image as an adjunct to fetal echo in t  CHD | 85 | Fetal CHD in echo requiring MRI | Weight >125 kg, claustrophobia, CI to MRI, no consent (16 did not qualify for MRI) | 32 |
| Manganaro 2009^38^ | Observational:  Sep 2007 to Aug 2008 | Feasibility to visualize normal and abnormal fetal cardiac structures using SSFP | 84  (43 with CHD and 40 without) | Fetal CHD in echo | - | 29 |
| Ryd 2021^39^ | Observational:  Jan 2017 to Jun 2020 | To evaluate the added diagnostic and prognostic value of CMRI in suspected fetal CHD | 31 | Incompletely visualized cardiovascular anatomy by fetal echo | - | 36 (median) |

**Table S5. Extracted methodological characteristics of the index and reference standard tests that were used to assign risk-of-bias and applicability concerns according to QUADAS-2.**

| **Author, Year** | **Index test: Fetal Cardiac MRI** | | | | **Reference test:**  **Postnatal diagnosis modality** | **MRI reporting:**  **Blinding to reference test** |
| --- | --- | --- | --- | --- | --- | --- |
|  | **Setting** | **Tesla, techniques, and gating** | **Qualified personnel** | **Timing of the index test** |  |  |
| Dong 2020^36^ | Pediatric Cardiology & Radiology Units, Teaching Hospital, China | # 1.5 T, two different generations of scanners during the study period  # cine MR sequences using SSFP with real-time SSFP, SSTSE, and non-gated phase contrast sequences with 2D reconstruction and motion correction | Two pediatric and fetal cardiac radiologists with 15 years of experience | Second trimester | Imaging, surgery | Likely |
| Goncalves 2022^15^ | Pediatric Cardiology & Radiology Units, Teaching hospital, USA | # 1.5 T (for fetuses with suspected isolated CHD) or 3T (for fetuses with suspected CNS anomalies)  # cine-MR sequences using balanced TFE sequence (bTFE), gated with fetal ECG | Two pediatric radiologists with 5-10 years' experience in fetal MRI | Third trimester | Echo, CT, surgery, autopsy | Likely |
| Hergert 2024^35^ | Fetal Medicine and Radiology units, Teaching hospital, Germany | # 3T  # cine MRI sequences using TSE & SSFP, DUS gated; performed under maternal breath hold. | Two radiologists with limited experience in fetal CMRI | Third trimester | Echo | Yes |

Table S5. Continued

| **Author, Year** | **Index test: Fetal Cardiac MRI** | | | | **Reference test:**  **Postnatal diagnosis modality** | **MRI reporting:**  **Blinding to reference test** |
| --- | --- | --- | --- | --- | --- | --- |
|  | **Setting** | **Tesla, techniques, and gating,** | **Qualified personnel** | **Timing of the index test** |  |  |
| Lloyd 2016^37^ | Pediatric Cardiology and Radiology units, Teaching hospital, UK | # SSFSE and balanced SSFP multi-slice imaging, non-gated | Two observers with expertise in congenital cardiac MRI | Third trimester | Echo, CT, MRI, surgery | Likely |
| Lloyd 2019^40^ | Pediatric Cardiology and Radiology units, Teaching hospital, UK | # 1.5T  # SSFSE, multi-slice 2D; 3D reconstruction with open software | Two observers with expertise in congenital cardiac MRI, and reviewed by 5 other pediatric cardiologists | Third trimester | Echo, CT, MRI, cardiac catheterization, surgery | Yes |
| Manganaro 2009^34^ | Pediatric Cardiology & Radiology Units, Teaching hospital, Italy | # 1.5T  # static phase using true-FISP SSFP and dynamic cine MRI using true-FISP 2D, non-gated | Two obstetric radiologists with longstanding experience | Third trimester | Echo, autopsy | Likely |
| Ryd 2021^39^ | Pediatric Cardiology and Radiology Units, Teaching Hospital, Sweden | # 1.5T  # Cine MRI using balanced SSFP, gated with DUS or self-gated | Three with extensive experience in fetal cardiology, echo, and CMR | Third trimester | Echo, CT, MRI, surgery, autopsy | Yes |

Figure S1. Summary statistics of pooled fetal cardiac MRI descriptions in the diagnosis of complex heart defects: (a) forest plots, and (b) hierarchical summary receiver operating characteristic curves.

Figure S2. Summary statistics of pooled fetal cardiac MRI descriptions in the diagnosis of septal defects and valvular anomalies: a) forest plots, and (b) hierarchical summary receiver operating characteristic curves.

Figure S3. Summary statistics of pooled fetal cardiac MRI descriptions in the diagnosis of aorta and pulmonary arterial anomalies: a) forest plots, and (b) hierarchical summary receiver operating characteristic curves.

Figure S4. Forest plots of pooled fetal cardiac MRI descriptions in the diagnosis of aberrant thoracic vessels.
