# Supplementary material for: Diagnostic accuracy of fetal cardiac magnetic resonance imaging in cardiac and thoracic vascular anomalies: a meta-analysis
Source: BMC Med Imaging. 2026 Jun 23;26:530. doi: 10.1186/s12880-026-02521-x (PMC13681847; doi:10.1186/s12880-026-02521-x)
Supplement: Supplementary file 4 [file 12880_2026_2521_MOESM4_ESM.docx]

**Supporting Information S4.**

Sensitivity analysis was done using the R package with a 0.5 continuity correction.

**1. Summary forest plots and summary receiver operating characteristic curves of pooled fetal cardiac MRI descriptions in the diagnosis of cardiac and thoracic vascular anomalies.**

**2. Summary forest plots and summary receiver operating characteristic curves of pooled fetal cardiac MRI descriptions in the diagnosis of complex heart defects.**

**3. Summary forest plots and summary receiver operating characteristic curves of pooled fetal cardiac MRI descriptions in the diagnosis of septal defects and valvular anomalies.**

**4. Summary forest plots and summary receiver operating characteristic curves of pooled fetal cardiac MRI descriptions in the diagnosis of aorta and pulmonary arterial anomalies.**

**5. Summary forest plots and summary receiver operating characteristic curves of pooled fetal cardiac MRI descriptions in the diagnosis of aberrant thoracic vessels.**

**Publication bias: Diagnostic odds ratio estimates of individual studies**

| Author | DOR (95% CI) |
| --- | --- |
| Dong 2020 | 309.06 (130.21, 733.58) |
| Goncalves 2022 | 972.63 (127.34, 7428.97) |
| Hergert 2024 | 36.39 (1.96, 677.40) |
| Lloyd 2016 | 75.29 (3.46, 1636.46) |
| Lloyd 2019 | 66.30 (3.00, 1464.41) |
| Manganaro 2009 | 861.00 (75.14, 9865.63) |
| Ryd 2021 | 13.43 (1.52, 118.63) |

The number of studies (k=7) is too small to test for small study effects

**Summary statistics of diagnostic accuracy of fetal cardiac MRI in the diagnosis of structural cardiac and thoracic vascular anomalies.**

| **Data** | **Sensitivity**  **(95% CI)** | **Specificity**  **(95% CI)** | **Correlation of sensitivity & false positive rates**  **(95% CI)** | **Positive likelihood ratio**  **(95% CI)** | **Negative likelihood ratio**  **(95% CI)** | **Diagnostic odds ratio**  **(95% CI)** | **Area under the curve**  **(95% CI)** |
| --- | --- | --- | --- | --- | --- | --- | --- |
| Pooled cardiac and thoracic anomalies | 0.79  (0.65, 0.88) | 0.97  (0.92, 0.99) | 0.37  (-0.53, 0.88) | 36.1  (9.85, 96.10)  Median: 30.3 | 0.22  (0.12, 0.36)  Median: 0.22 | 167.00  (45.00, 440.00)  Median: 141.00 | 0.95 |
| *Sub-groups* |  |  |  |  |  |  |  |
| Complex heart defects | 0.88  (0.79, 0.94) | 0.99  (0.98, 1.0) | 0.35  (-0.55, 0.87) | 149.00  (39.00, 402.00)  Median: 125.00 | 0.13  (0.07, 0.21)  Median: 0.12 | 1240.00  (320.00, 3350.00)  Median: 1040.00 | 0.98 |
| Septal defects and valvular lesions | 0.69  (0.44, 0.86) | 0.99  (0.97, 1.0) | 0.06  (-0.73, 0.78) | 61.50  (25.50, 119.00)  Median: 57.30 | 0.33  (0.14, 0.57)  Median: 0.32 | 210.00  (70.80, 489.00)  Median: 186.00 | 0.98 |
| Aorta and pulmonary vessel lesions | 0.75  (0.53, 0.89) | 0.99  (0.94, 1.0) | -0.13  (-0.80, 0.69) | 68.60  (13.20, 218.00)  Median: 52.80 | 0.27  (0.11, 0.48)  Median: 0.26 | 303.00  (40.80, 1110.00)  Median: 213.00 | 0.96 |
| Aberrant vessels | 0.69  (0.54, 0.82) | 0.99  (0.98, 1.0) | -0.15  (-0.74, 0.57) | 142.00  (37.10, 384.00)  Median: 119.00 | 0.31  (0.19, 0.47)  Median: 0.31 | 479.00  (110.00, 1370.00)  Median: 389.00 | 0.94 |
